# Treatment satisfaction with disease-modifying therapy is the only predictor of Adherence among multiple sclerosis patients from Upper Egypt

**DOI:** 10.1038/s41598-024-57116-9

**Published:** 2024-03-25

**Authors:** Eman M. Khedr, Doaa M. Mahmoud, Hussein B. Hussein, Islam E. L. Malky, Sarah S. Mostafa, Ayman Gamea

**Affiliations:** 1 Department of Neuropsychiatry, Faculty of Medicine, Assiut University Hospital, Asyût, Egypt; 2 Neuropsychiatric Department, Faculty of Medicine, Aswan University Hospital, Aswân, Egypt; 3 Neuropsychiatric Department, Faculty of Medicine, South Valley University, Qena University Hospital, Qena, Egypt

**Keywords:** Multiple sclerosis, Disease-modifying treatment, Non-adherence, Treatment satisfaction, Depression, Fatigue, Sleep, Immunology, Diseases, Neurology

## Abstract

Despite the proven efficacy of the disease-modifying therapy (DMT) for multiple sclerosis (MS), the rates of non-adherence are frequently high. We aimed to evaluate the rate of non-adherence to the first DMT in Upper Egypt and identify different contributing factors. Out of 310 patients, ninety-seven adult patients with RRMS were recruited from three MS units located in Upper Egypt and were subjected to the following: complete clinical history, expanded disability status score (EDSS), Eight-item Morisky Medication Adherence Scale (MMAS-8), abbreviated Treatment Satisfaction Questionnaire for Medication-9 (TSQM-9), Hamilton depression scale, Fatigue Severity Scale (FSS) and the Pittsburgh Sleep Quality Index (PSQI). According to MMAS-8 scores, 63 (64.9%) of patients were non-adherent to their first DMT. Non-adherent patients are more likely to have longer disease duration (*p* = 0.002), longer duration on first DMT (*p* = 0.030), first DMT-start date before 2019 (*p* = 0.040), and lower treatment satisfaction scores (*p* = 0.016). However, there was no significant relation with physical disability, depression, fatigue, or sleep quality. On the regression analysis model, a lower treatment satisfaction score was the only predictor of DMT non-adherence (*p* = 0.012). Despite expanding DMT options, non-adherence among MS patients in Upper Egypt is high. Treatment satisfaction with DMT is the only predictor of adherence among MS patients of Upper Egypt. Adherence and satisfaction with the prescribed DMT should be assessed carefully to maximize DMT benefits.

## Introduction

Multiple sclerosis (MS) is one of the leading causes of disability among young and middle-aged adults with consequent loss of productivity^1^. Even though it is an incurable disease, a breakthrough has been made during the last 20 years in the industry of disease-modifying treatments (DMTs), that altered the disease course by either lowering disease activity or slowing down disability accumulation^2^. However, like many other chronic disorders—despite the proven benefits of treatment—the rates of DMT adherence among remitting relapsing MS (RRMS) patients are often quite low^3^.

For patients with MS, non-adherence could lead to specific consequences including the risk of relapse, disability accumulation, and disease progression. Moreover, from the economic point of view, increases the need for hospitalization, more utilization of healthcare resources, and subsequently the need for higher-cost DMTs for the progressive course^4–6^.

Although non-adherence could result from conditions out of the patient’s control like forgetfulness or difficulty obtaining the drug, it could also be intentional resulting from patient dissatisfaction due to the negative perception of drug efficacy, tolerability, or even disease prognosis^7^. As such, targeting the intentional causes of non-adherence during patient-physician communication could improve adherence rates to achieve the maximal benefits of the prescribed drug^7^.

Since patients are expected to live with MS for about thirty or forty years^8,9^, improvements in adherence have the potential to improve patient's clinical outcomes and lower the economic burden^10^. In a developing country with scarce resources like Egypt particularly in the South, early addressing and targeting intentional causes of non-adherence, could be an important therapeutic goal.

## Results

Ninety-seven patients with RRMS were recruited from 3 different MS units located in the south of Egypt. Sixty-nine patients (71.1%) were females with a female-to-male ratio of 2.5:1. The median age was 32.0 years (IQR:14) while the median age at onset was 27.0 years (IQR:12). The duration of illness has ranged from 18 months up to 13 years with a median duration of 6 years (IQR: 5.5 years). The median number of relapses experienced during the course of illness was 4 (IQR: 2). The median EDSS score at the time of presentation was 3 (IQR: 2.5).

Seven different DMTs were identified and were categorized into 3 groups according to their routes of administration (Self-injectables “*Interferon beta*”, Orals “*Fingolimod- Teriflunomide- Dimethyl fumarate*” and Infusions “*Ocrelizumab and Rituximab”).* Table 1 shows the differences in DMT utilization before and after 2019. According to MMAS-8, 34 patients (35.1%) were considered DMT-Adherent to the first prescribed DMT (8 score on MMAS-8) and 63 patients (64.9%) were non-adherent to the first prescribed DMT (MMAS-8 score < 8).

**Table 1:** Comparison between the frequency of different DMTs according to the route of administration before and after the year 2019 (n = 97).

	Before 2019	2019 and after
Self-Injectable DMTs, n (%)	26.0 (78.8)	36.0 (56.3)
Oral DMTs, n (%)	7.0 (21.2)	22.0 (34.4)
Infusion DMTs, n (%)	0.0 (0.0)	6.0 (9.4)

The sociodemographic and clinical differences between the adherent and non-adherent groups are shown in Table 2*.* No significant differences between groups, as regards demographic factors like sex, age, marital status, and level of education, were recorded. When the clinical variables were evaluated, the duration of the disease was significantly longer in the non-adherent than adherent group. Other clinical factors like age at onset, relapse frequency, EDSS score, and comorbid conditions like depression, fatigue, and sleep disorders did not differ significantly between groups.

**Table 2:** Socio-demographic and clinical characteristics of MS patients according to their medication adherence to the first DMT (n = 97).

	Adherent (MMAS = 8) (n = 34)	Non-adherent (MMAS < 8) (n = 63)	*p*-Value
Age at presentation, Median (IQR)	35.0 (18.0)	31.0 (14.0)	0.788
Sex, n (%)			0.135
Male	13.0 (38.2)	15.0 (23.8)	
Female	21.0 (61.8)	48.0 (76.2)	
Marital status, n (%)			0.160
Single	15.0 (44.1)	19.0 (30.2)	
Married	18.0 (52.9)	41.0 (65.1)	
Divorced	1.0 (2.9)	2.0 (3.2)	
Widow	0.0 (0.0)	1.0 (1.6)	
Level of education, n (%)			0.179
Low-level education	3.0 (8.8)	13.0 (20.6)	
Basic level education	16.0 (47.1)	29.0 (46.0)	
High-level education	15.0 (44.1)	21.0 (33.3)	
Age at onset, Median (IQR)	32.0 (19.0)	26.0 (11.0)	0 .093
Disease duration in years, Median (IQR)	4.0 (2.3)	6.0 (4.5)	0.002*
Relapse frequency, Median (IQR)	3.0 (2.0)	3.0 (3.0)	0.074
EDSS, Median (IQR)	4.5 (3.5)	2.5 (2.0)	0.915
Depression, n (%)	22.0 (64.7)	42.0 (66.7)	0.846
Fatigue, n (%)	26.0 (76.5)	55.0 (87.3)	0.170
Sleep disorder, n (%)	14.0 (41.2)	38.0 (60.3)	0.071
Year of initiation of first DMT, n (%)			0.040*
Before 2019	7.0 (20.6)	26.0 (41.3)	
2019 and after	27.0 (79.4)	37.0 (58.7)	
First DMT rout of administration, n (%)			0.241
Injectables	19.0 (55.9)	43.0 (68.3)	
Oral	11.0 (32.4)	18.0 (28.6)	
Infusions	4.0 (11.8)	2.0 (3.2)	
Duration of 1st DMT, Median (IQR)	2.0 (2.0)	4.0 (3.0)	0.030*
1st DMT Treatment satisfaction (TSQM-9), Median (IQR)			
Efficiency	77.8 (19.5)	66.7 (33.3)	0.028*
Tolerability	75.0 (29.2)	61.1 (38.9)	0.054
General satisfaction	77.8 (36.1)	63.9 (55.6)	0.052
Total TSQM-9 score	73.9 (26.8)	62.1 (33.3)	0.016*

IQR: Interquartile Range.

p: p-value for comparing the two studied groups.

*: Statistically significant at p ≤ 0.05.

Although the route of first DMT administration did not differ significantly between the adherent and non-adherent group, however, the year of DMT initiation was significant, where patients initiating their first DMT in recent years (≥ 2019) tend to be more adherent than those initiating treatment in former years (before 2019). Also, the duration of the 1st DMT was significantly different between both groups, where the non-adherent patients were more likely to have a longer duration on the 1st DMT. Regarding treatment satisfaction with 1st DMT, scores for the efficacy, as well as the total score of the TSQM-9 were significantly higher in adherent than non-adherent groups of patients.

When Non-adherence to the 1st DMT was considered as an outcome variable, logistic regression analysis shows that the total score of TSQM-9 was the only factor that might predict non-adherence (odds ratio [OR] 0.970; 95% confidence interval [CI]: 0.948–0.993; *P* = 0.012) as shown in Table 3.

**Table 3:** Univariate and multivariate Logistic regression analysis for the parameters affecting treatment non-adherence.

	Univariate		^#^Multivariate	
	*p*	OR (95% C.I)	*p*	OR (95% C.I)
Disease Duration	0.006*	1.229 (1.061–1.423)	0.055	1.239 (0.995—1.543)
Duration of the First DMT	0.103	1.219 (0.961–1.545)		
Year of first DMT initiation	0.044*	0.369 (0.140–974)	0.933	0.938 (0.209—4.004)
Before 2019				
2019 and after				
The total score of the first DMT TSQM-9	0.018*	0.974 (0.954—0.996)	0.012*	0.970 (0.948—0.993)

OR: Odd`s ratio.

C.I, Confidence interval; LL, Lower limit; UL, Upper Limit.

^#^All variables with *p* < 0.05 were included in the multivariate.

*Statistically significant at *p* ≤ 0.05.

Out of the 97 studied patients, 40 (41.2%) of patients had already switched to a 2nd DMT at the time of the study. Those patients were evaluated for their adherence and treatment satisfaction with the first and second DMTs. Only 13/40 patients (32.5%) were adherent to the 1st DMT compared to 28/40 (70.0%) who were adherent to the 2nd DMT. Also, treatment satisfaction was found to be statistically significantly higher in the 2nd DMT as shown in Table 4.

**Table 4:** Comparison between adherence and satisfaction in the first DMT and second DMT in the switcher group of patients (n = 40).

	1st DMT	2nd DMT	*P*
Adherence, n (%)	13.0 (32.5%)	28.0 (70.0%)	< 0.001*
Efficiency, Median (IQR)	55.6 (43.0)	83.3 (15.3)	< 0.001*
Tolerability, Median (IQR)	58.7 (36.1)	83.3 (15.2)	< 0.001*
General satisfaction, Median (IQR)	41.7 (53.4)	86.1 (16.6)	< 0.001*

IQR: Interquartile Range.

p: *p*-value for comparing the two studied groups.

*Statistically significant at *p* ≤ 0.05.

## Discussion

The landscape of DMT utilization has significantly changed over time in all countries as oral DMTs were gradually introduced. In Egypt only in the most recent years from 2019 and onwards, a substantial increase in the utilization of oral and infusion treatments was evident in comparison with the oldest time periods when injectable DMTs were dominated^11^. When choosing the proper DMT, the physician puts the main focus on the efficacy and safety of the drug. However, since non-adherence to the prescribed DMTs even the most powerful drugs is associated with reduced efficacy, in this particular era of expanding DMT options, patient’s preferences and satisfaction with their medication will affect adherence and therefore the treatment outcomes^12^. Therefore, a good understanding and early management of these intentional factors associated with DMT non-adherence may help patients with MS adhere to their DMT.

The main finding of the present study is the low rate of adherence (35%) to the first prescribed DMT. Adherent patients had a shorter disease duration, a shorter duration of DMT utilization, and greater treatment satisfaction than non-adherent patients.

When reviewing the literature, the rates of adherence were variable among different studies. Over a two-year period, Hansen et al.^13^ found that only 30–40% of patients with MS (pwMS) were adherent to their treatment. In a study from Medina, Saudi Arabia^14^, about 37.8% of the studied patients were considered to be adherent to DMTs. While another study also from Saudi Arabia^15^ (using MMAS-8; the same scale used in our study) found a very low rate of adherence (9.7%). In Argentina, using the mean positional ratio during a telephone interview, a higher rate of adherence was reported (47.7%)^16^. A Brazilian study by Camera and Gondim identified an adherence rate of 46% according to the (MMAS-8),^17^. In contrast, other studies reported very high rates of DMT adherence among pwMS up to 88%^5,18–22^. This wide range of the reported adherence rates could be explained by variability in the definitions of adherence across different studies and the response bias in self-reported measures^23^.

In the present study, we did not find any differences in the sociodemographic factors of the studied sample, in relation to DMT adherence, although several studies had shown that men had better treatment adherence than women^24–26^.

When factors related to the disease nature were evaluated in relation to adherence, no significant relationship was found between relapse frequency (reflecting the drug efficacy) and non-adherence. However, the disease duration was significantly shorter in the adherent group than non-adherent group. A similar observation was noted by Devonshire et al.^27^. Furthermore, adherent participants had also been taking their current DMTs for a shorter period of time than non-adherent participants predicting that the duration of DMT utilization itself might be a predictive factor for adherence. In line with the previous finding, McKay et al.^28^ found that disease duration of more than five years was significantly associated with non-adherence. The association of non-adherence with increased disease duration could be partially explained by the progressive phase of the disease in which most first-line DMTs lose their efficacy with consequent loss of persistence to the prescribed DMT. However, we couldn’t find a similar association between adherence and EDSS scores.

In the current study, 65% of patients were found to be depressed on HAM-D scale, however, no statistically significant association was found as regards adherence. Besides the association of depression with non-adherence in several studies, it is considered a common side effect of DMTs^24,25,29,30^.

As regards Fatigue, although the prevalence was very high (83%), no significant association was found between fatigue and adherence. The same was observed in the study from Argentina^31^ in contrast to other studies that showed a relationship between fatigue and non-adherence^29,32^.

Effect of DMTs on sleep quality have been reported as important contributors of poor sleep with 47 to 62% of pwMS report sleep problems^33,34^. In the current study, according to PSQI about 53% of patients were considered to have poor sleep quality. Despite the high prevalence of sleep disturbances in our cohort, no significant association was found regarding its effect on adherence, and on reviewing the literature we could not find studies that examined the direct relation of sleep disturbance and DMT adherence.

A link between medication adherence in pwMS and treatment satisfaction was evident^12^. To objectively evaluate treatment satisfaction, the TSQM was used, not only because it was validated in several chronic diseases including MS, but also being available in the Arabic language and is easy to administer^35^. The mean scores of treatment satisfaction were significantly higher in the adherent patients compared to the non-adherent in the current study. These findings are in accordance with those reported by several studies, as adherent patients usually report greater satisfaction with their DMTs, particularly as regards convenience and effectiveness^12,36–38^. One best methods to demonstrate the role of treatment satisfaction on adherence was to compare the changes in adherence and treatment satisfaction in patients who switched to another DMT. In the current study, forty-one switchers were identified in whom treatment satisfaction scores have raised significantly after the second DMT switch with improved adherence rate. A study by Calkwood et al. showed improved scores on the sfficacy, side effects, and convenience scales in the switchers from injectable DMTs to fingolimod versus patients staying on the injectables^39^. Therefore, it is recommended to use TSQM as a tool to screen for patients who may become non-adherent due to unsatisfactory components of their medication.

Despite many studies where the most shared factor was the type of DMT particularly the route of administration^16^, in the current study, being on injectable DMTs did not differ significantly between adherent and non-adherent groups. However, we found that the year of initiation of the first DMT (of different routes of administration) had statistical significance, where patients who recently initiated DMT in 2019 and onwards are more likely to be adherent than those who started earlier, indicative for the health-related quality of care with increased numbers of MS units around the country. In contrast to the observation that was reported from a Canadian study where subjects who initiated therapy in earlier years were less likely to be non-adherent or to discontinue their DMT within the first 12 months than those who started treatment in recent years. However, the study did not identify any specific characteristics associated with adherence to the DMTs^21^.

Lack of medication adherence remains a challenge among pwMS. Maximizing adherence to DMTs is an important therapeutic goal that will enable patients to gain the full benefit from their treatment with improved clinical outcomes and reduction of medical resource utilization^40^. We found that only patient satisfaction with DMT could predict their adherence and persistence with treatment. We recommend practicing the informed shared decision between MS specialist and patient while choosing the first DMT. Considering patient convenience alongside efficacy and safety will definitely make the patient more willing to adhere to the prescribed DMT. Regular follow-up of patients to objectively screen for non-adherence and non-satisfaction will help early catch and management of suboptimal treatment responses.

The current study has some limitations such as the relatively small sample size of patients, lacking MRI lesion load, and the access to healthcare facilities (for example the availability of DMTs and facilities for infusion drugs) all may contribute to non-adherence. Future studies including these points are recommended.

## Methods

### Participants

Patients were consecutively recruited from three MS centers located in Upper Egypt: Assiut, South Vally, and Luxor during one year from January 1st till 31st December 2022. All patients with RRMS diagnosed according to the 2017 McDonald’s criteria^41^ who received any DMT for at least one year were eligible to participate in the study. Inclusion criteria were: Patients ≥ 18 years old and both sexes were included. Patients were excluded if there was any of the following: (a) Diagnosis of demyelinating diseases other than MS, (b) patients diagnosed with progressive MS (SPMS or PPMS), (c) cognitive impairment diagnosed with the Arabic version of the brief international cognitive assessment for multiple sclerosis (BICAMS) as 1.5 SD below the mean scores of a control group (cutoff scores were: 22 for SDMT, 38 for CVLT, and 10 for BVMTR)^42^, (d) Presence of other systemic disorder or being on any long-term therapy, (e) Incomplete clinical or radiological data were provided, and f) Subject refused to participate or to provide written informed consent.

### Study procedures

**Figure 1:**
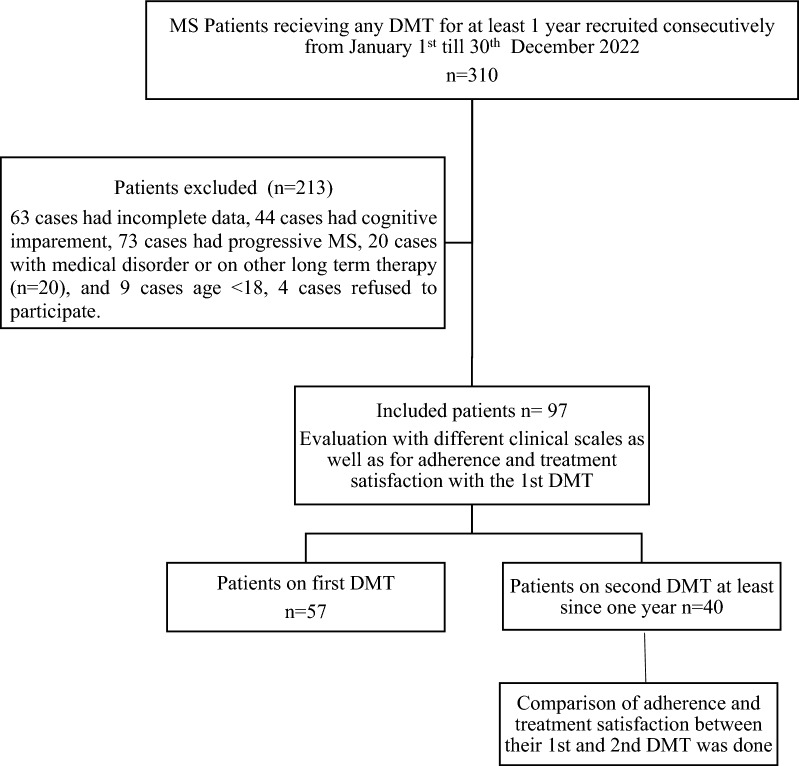
Study design shows the total number of the MS patients during the period of the study was 310 patients 213 were excluded and only 97 RRMS were participated in the study.

This was a cross-sectional, multicentric study (Fig. 1: Flowchart of study design). Each patient after fulfilling the inclusion and exclusion criteria was submitted to the following:

1. Complete sociodemographic and clinical data including age, level of education, marital status, age at onset, duration of disease, number of relapses, and therapeutic history.
2. Disease severity was assessed using the Expanded disability status score (EDSS).
3. Patients’ medication adherence was objectively evaluated using the Arabic version of the eight-item Morisky Medication Adherence Scale (MMAS-8)^43,44^. The MMAS-8 consists of 8 questions, with the first 7 having Yes or No replies to prevent acquiescence bias and the last question having an answer on a scale of 5 points ranging from low to high levels of adherence. Using the usual scoring criteria, between 0 and 8 make up the total summated adherence score. A score of less than six was seen as low adherence, six to seven was regarded as medium adherence, and eight was regarded as strong adherence. In the current study, patients were classified as Adherent (MMAS-8 score of 8) or Non-Adherent (MMAS-8 score of < 8).
4. Satisfaction with the first prescribed DMT was assessed using the abbreviated Arabic version of the Treatment Satisfaction Questionnaire for Medication (TSQM-9)^45,46^. It consists of three blocks of questions evaluating the patient's subjective experience with treatment's effectiveness, tolerability, and overall satisfaction. By summing the obtained scores and calculating the percent satisfaction score, greater scores indicated better satisfaction. After using the scale scoring algorithm, each TSQM subscale may have a value between 0 and 100, with higher scores indicating better satisfaction.
5. Depression was evaluated using the Arabic version of the Hamilton depression rating-17-item version (HAM-D 17)^47,48^: Based on the patient's conduct during the interview, scores on three items—insight, psychomotor agitation, and retardation—are calculated. Patients with scores of 8 or more were depressed (8–16: mild depression, 17–23: moderate depression, and more than 24: severe depression).
6. Fatigue was evaluated using the Arabic version of the Fatigue Severity Scale (FSS)^49,50^: The FSS is a 9-item self-reported scale. Eight questions focus on the physical effects of exhaustion and how it affects daily tasks, while one question asks about the cognitive effects of fatigue. FSS scores are the mean^1–7^, where patients with scores of 4 or more are considered fatigued.
7. The quality of sleep was assessed as a possible contributing factor using the Arabic version of the Pittsburgh Sleep Quality Index (PSQI)^51,52^. This scale measures sleep quality during the previous month and can be used to distinguish between “excellent” and “bad” sleepers, detect nightly sleep problems, and track the development of sleep disorders. It consists of five additional bed partner questionnaires in addition to the 19 self-rated quizzes. On a scale from 1 to 21, it delivers a global sleep score; higher scores suggest more sleep complaints (≥ 5 indicates a sleep problem).
8. Patients on 2nd DMT at the time of the study ^40cases^ were evaluated for their adherence and treatment satisfaction and subgroup analysis was done to compare their 1st DMT versus 2nd DMT using MMAS-8 and TSQM-9.

### Ethics

This study was approved by the Local Ethics Committee of Faculty Medicine, South Valley University (IRB no: SUV MED NAP020220756) and was conducted in accordance with the provisions of the Declaration of Helsinki. Also, written informed consent from all participants was obtained after the description of the aim of the study and methods before participation in the study.

### Statistical analysis of data

The data was analyzed using the 26.0 Version of the IBM Statistical Package for Social Sciences software (Armonk, NY: IBM Corp). Ratio and percentage were used to describe the qualitative factors. The Shapiro–Wilk test was used to examine the normality of the data distribution. The mean and standard deviation were used to describe quantitative data with normal distribution, whereas the median and mid-quartile range were used to describe those with non-normal distribution.

Adherence was analyzed as a dichotomous variable and categorized as “adherent” or “non-adherent” according to the MMAS-8 score (MMAS of 8 was considered adherent while < 8 was considered non-adherent). A bivariate association analysis was done with different variables, followed by a univariate logistic regression analysis for significantly associated factors. Then all significant predictor variables were simultaneously entered into the multiple logistic regression model using non-adherence as an outcome variable. Patients on 2nd DMT at the time of the study underwent subgroup analysis to compare adherence and treatment satisfaction between the 1st DMT and 2nd DMT using McNemar and Wilcoxon signed rank sum tests respectively. A cutoff point of 0.05 was used for statistical significance.

## Data Availability

Data can be made available to qualified investigators upon reasonable request to the corresponding author.
